# Procalcitonin levels in fresh serum and fresh synovial fluid for the differential diagnosis of knee septic arthritis from rheumatoid arthritis, osteoarthritis and gouty arthritis

**DOI:** 10.3892/etm.2014.1870

**Published:** 2014-07-29

**Authors:** CHENGGONG WANG, DA ZHONG, QIANDE LIAO, LINGYU KONG, ANSONG LIU, HAN XIAO

**Affiliations:** Department of Orthopedics, Xiangya Hospital, Central South University, Changsha, Hunan 410008, P.R. China

**Keywords:** differential diagnosis, procalcitonin, septic arthritis, fresh serum, fresh synovial fluid

## Abstract

Whether the levels of procalcitonin (PCT) in the serum and synovial fluid are effective indicators for distinguishing septic arthritis (SA) from non-infectious arthritis remains controversial. The present study aimed to evaluate whether PCT levels in fresh serum or fresh joint fluid may be used in the differential diagnosis of SA from rheumatoid arthritis (RA), osteoarthritis (OA) and gouty arthritis (GA). From January 2012 to June 2013, 23 patients with knee SA, 21 patients with RA, 40 patients with OA and 11 patients with GA were enrolled in the current study. The levels of PCT were measured within 24 h after specimen collection at room temperature. An enzyme-linked fluorescence assay (ELFA) was used to detect the levels of PCT in the serum and synovial fluid. The correlations between the levels of PCT in the serum and synovial fluid and the arthritic patient groups were determined by the Nemenyi test. Areas under the receiver operating characteristic (ROC) curve were calculated to evaluate the accuracy of the correlations. The levels of PCT in the serum and joint fluid of the patients in the SA group were higher compared with those of the other groups (P<0.01) and there were no significant differences among the RA, OA and GA groups in these levels. A PCT level of <0.5 μg/l in the serum and synovial fluid had high specificity in the differential diagnosis of SA from RA, OA and GA. Synovial fluid PCT revealed significantly greater sensitivity than serum PCT. The accuracy of the differential diagnosis of SA by the serum levels of PCT was significantly lower than that by the synovial fluid levels of PCT. The levels of PCT in the serum and synovial fluid may be used as alternative laboratory indicators to distinguish between SA and the non-infectious types of arthritis; however, the PCT levels in fresh synovial fluid are more sensitive and accurate indicators than PCT levels in fresh serum.

## Introduction

The incidence rate of septic arthritis (SA) has been increasing over the last few years. Early diagnosis as well as a prompt and effective treatment are vital in order to avoid severe outcomes (1). However, the clinical manifestation of SA is similar to that of non-septic arthritis, including rheumatoid arthritis (RA), osteoarthritis (OA) and gouty arthritis (GA), which causes difficulty in the differentiation of SA from RA, OA and GA in clinics. Currently, the detection of bacteria in synovial fluid culture remains the primary index for the diagnosis of SA. However, this has a number of disadvantages including aseptic surgery, a long turn-around time and the potential generation of a false positive or negative result (2,3). Prior to the bacterial culture results becoming available, several other clinical tests, including X-ray imaging (4), routine blood tests (5), erythrocyte sedimentation rate (ESR) measurements (6), synovial fluid white blood cell (WBC) count (7) and levels of CRP (C reactive protein) (8,9) may be used for SA diagnosis. However, none of these tests are sensitive enough to produce an accurate diagnosis and they frequently lead to misdiagnosis and/or the delay of treatment. For example, CRP is another positive predicator in the diagnosis of SA (10–14); however, the diagnostic accuracy of CRP is affected by steroids (15). A quick and sensitive test is thus required.

Procalcitonin (PCT), a precursor of the calcitonin peptide produced due to bacterial endotoxins, tumor necrosis factor (TNF)-α and interleukin (IL)-6 (16,17), is a novel predictor for the diagnosis of bacterial infection (18,19). As a secondary inflammatory factor, PCT is not directly involved in initiating the septic process but may enlarge and aggravate the pathological process of sepsis (20) and is unresponsive or only mildly reactive to aseptic inflammation and viral infection (21). PCT has been used in the diagnosis of systemic infections or infectious shock (22–27). Unlike CRP, the diagnostic accuracy of PCT is not affected by steroids (28). A previous meta-analysis demonstrated that PCT was more accurate than CRP in diagnosing systemic bacterial infection, regardless of pathogen type (29). Despite the widespread use of PCT in the diagnosis of numerous systemic infectious diseases, including sepsis and pneumonia (30–33), the use of PCT in the diagnosis of SA, RA, OA and GA remains limited. Serum levels of PCT perform better than synovial fluid levels of PCT in the diagnosis of SA (34). However, PCT levels among non-infectious types of arthritis (OA, RA and GA) have not, to the best of our knowledge, been compared, although the level of PCT in SA has been compared with that of non-inflammatory arthritis in the current study. The synovial fluid levels of PCT have been reported to be positive indicators of SA (35). A previous study has demonstrated that the synovial fluid level of PCT is significantly higher in patients with SA than in patients with OA, and that CRP levels differ significantly among patients with SA, RA and OA (36). The serum level of PCT is a specific marker of SA but with a low sensitivity; serum PCT in association with CRP has not been useful for the diagnosis of SA (37).

Thus, the applicability of using the levels of PCT in the serum or synovial fluid as effective indicators for distinguishing SA from non-infectious forms of arthritis remains controversial. A study involving patients with SA, RA, OA and GA would be instrumental in resolving the controversy. The discrepancy in the effectiveness of applying serum or synovial fluid PCT in diagnosis may result from the condition of the serum or synovial fluid samples. The specimens in certain previous studies (34–37) were frozen for longer than 24 h prior to the experiments being conducted while the half-life of PCT in the synovial fluid has been reported to be ~20–24 h (38). The present study aimed to evaluate whether serum or joint fluid levels of PCT may be used in the differential diagnosis of SA from RA, OA and GA using fresh serum and synovial fluid samples.

## Materials and methods

### Patients

A non-blind method was used for selection of participants in the present study and subjects volunteered to become study subjects. All subjects were selected from the outpatient service of Xiangya Hospital of Central South University (Changsha, China) from January 2012 to June 2013. The patients were selected according to the following criteria: i) Patients with various types of arthritis other than SA, RA, OA or GA were excluded from the present study. Diagnosis was carried out according to the criteria set by the American College of Rheumatology (ACR) and confirmed by bacterial culture of the patients’ synovial fluid. ii) The patients had not received any antibiotic or joint puncture treatments prior to enrollment in the current study. iii) Patients were excluded if the joint(s) being treated had been subjected to artificial joint surgery. iv) All patients included in the present study had been evaluated using the joint fluid bacterial culture test. Patients with SA were excluded if the result of their bacterial culture test was negative; RA, OA and GA patients were excluded if the result of their bacterial culture test was positive. v) Each patient provided informed written consent and approval from the Institutional Review Board (IRB) of the Xiangya Hospital was obtained prior to data extraction and analysis.

### Collection of specimens

A total of 5 ml blood was drawn from a vein at the elbow of each patient followed by centrifugation at 2,756 × g for 10 min. A synovial fluid sample ≥200 μl in volume was aseptically aspirated from the patellofemoral articular surface. Patients from which <200 μl synovial fluid was collected were excluded from the study. If the two knees of a patient exhibited symptoms of SA, RA, OA or GA, synovial fluid was only drawn from the knee with the more severe symptoms. All samples were stored at room temperature (10–25°C) prior to analysis.

### Determination of the levels of PCT in the serum and synovial fluid

Levels of PCT were tested within 24 h after serum and fluid collection at room temperature (10–25°C) by enzyme linked fluorescent analysis (ELFA) using a PCT quantitative determination kit and fluorescence reading machine (Vidas^®^ B.R.A.H.M.S PCT™; bioMérieux, Marcy l’Etoile, France) according to the manufacturers’ instructions.

### Statistical analysis

All statistical analyses were performed using SPSS software, version 19.0 (SPSS, Inc., Chicago, IL, USA), unless otherwise specified. P<0.01 was considered to indicate a statistically significant difference. Correlations of the serum and synovial fluid levels of PCT between groups were determined by the Nemenyi test. Areas under the receiver operating characteristic (ROC) curve were calculated to evaluate the accuracy of the correlations.

## Results

### Patient characteristics

A total of 95 patients were enrolled in the current study. Of these, 23, 21, 40 and 11 patients had SA, RA, OA and GA, respectively. The corresponding male/female ratios were 15/8, 6/15, 19/21 and 10/1, respectively, and the corresponding average ages were 46.6±3.6, 35.0±2.2, 66.2±2.6 and 60.8±5.6 years, respectively (Table I). The types and number of pathogenic bacteria of SA are also listed in the table.

### Levels of PCT in the serum and synovial fluid

**Figure 1:**
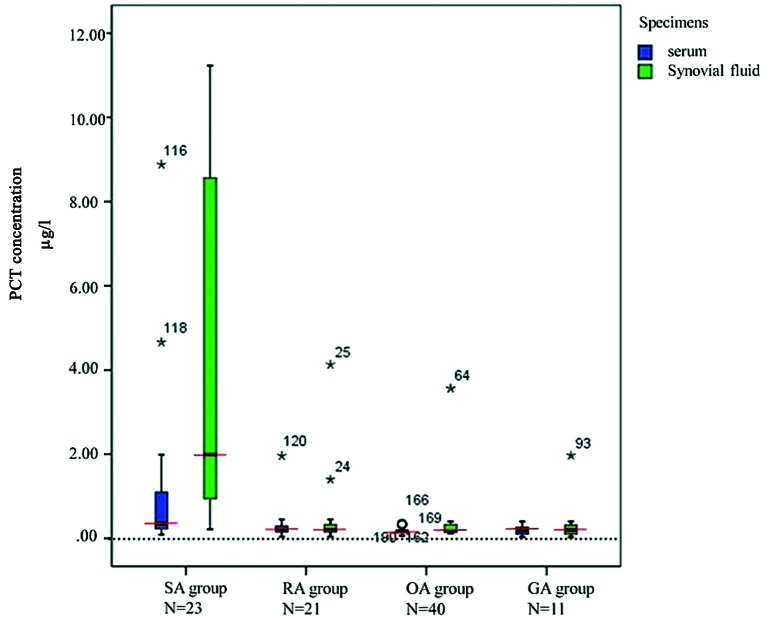
Serum and synovial fluid levels of procalcitonin (PCT) among patients with septic arthritis (SA), rheumatoid arthritis (RA), osteoarthritis (OA) and gouty arthritis (GA). Correlations of the serum and synovial fluid levels of PCT between each pair of patients with arthritis were determined by the Nemenyi method.

The levels of PCT in the serum and synovial fluid in the SA group were significantly higher compared with those in the other three groups (P<0.01). No significant differences in the levels of PCT in the serum or synovial fluid were observed among the RA, OA and GA groups (P>0.01; Fig. 1).

### Correlation between the levels of PCT in the serum and synovial fluid and SA, RA, OA and GA

The majority of the serum levels of PCT in the four groups (SA, 65.2%; RA, 100%; OA, 97.5% and GA, 100%) were <0.5 μg/l. Serum levels of PCT in ~34.8% of patients with SA were in the range 0.5–10.0 μg/l. The levels of PCT in the synovial fluid in the majority of patients in the RA, OA and GA groups were <0.5 μg/l (RA, 95.2%; OA, 95.0% and GA, 90.9%). The levels of PCT in the synovial fluid of 39.1% and 30.4% of patients in the SA group were 0.5–2 μg/l and 2–10 μg/l, respectively (Table II).

To differentiate SA from the forms of non-infectious arthritis, serum and synovial fluid levels of PCT <0.5 μg/l had great specificity, positive predictive value (PPV) and a negative predictive value (NPV). Synovial fluid levels of PCT <0.5 μg/l had significantly higher sensitivity compared with serum levels of PCT. Serum and synovial fluid levels of PCT at 2 or 10 μg/l revealed great specificity but low sensitivity (Table III).

### Accuracy of serum and synovial fluid PCT in discriminating SA from RA, OA and GA

**Figure 2:**
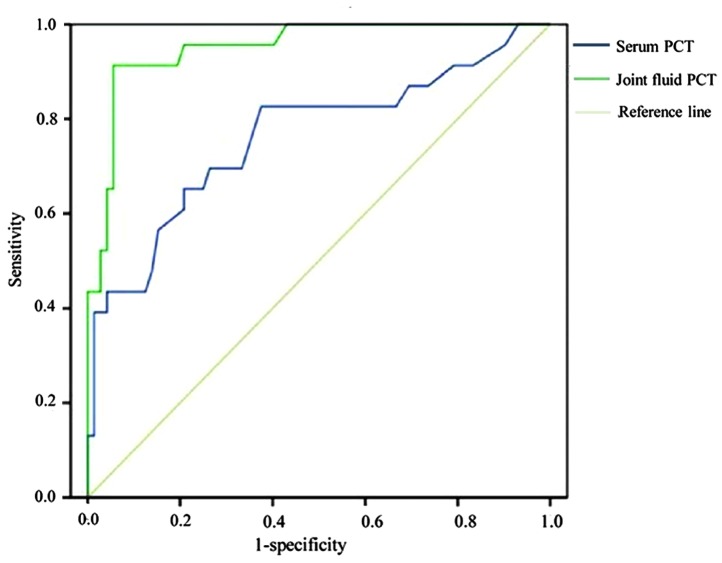
Receiver operating characteristic (ROC) curve of serum and synovial fluid levels of procalcitonin (PCT) in the differential diagnosis of septic arthritis (SA) from rheumatoid arthritis (RA), osteoarthritis (OA) and gouty arthritis (GA). The areas under the ROC curve of the serum and synovial fluid levels of PCT were calculated and the accuracy of using serum and synovial fluid levels of PCT in the differential diagnosis of SA from RA, OA and GA was analyzed.

The areas under the ROC curve of the serum and synovial fluid levels of PCT were calculated and the accuracy of serum and synovial fluid PCT in discriminating SA from RA, OA and GA was determined. The area under the ROC curve of the serum levels of PCT was 0.761 and the area under ROC curve of the synovial fluid levels of PCT was 0.951 (Fig. 2 and Table IV). The accuracy of serum PCT was significantly lower compared with that of synovial fluid PCT in discriminating SA from RA, OA and GA (P<0.01).

## Discussion

To investigate whether serum and/or joint fluid PCT can be used in distinguishing SA from RA, OA and GA, the levels of PCT in fresh serum and synovial fluid samples of patients diagnosed with SA, RA, OA or GA were measured. The present study demonstrated that the levels of PCT in the serum and synovial fluid samples from patients in the SA group were significantly higher compared with those in the RA, OA, or GA groups. Levels of PCT in the serum and synovial fluid <0.5 μg/l had high specificity in the differential diagnosis of SA from RA, OA and GA. The accuracy of the differential diagnosis of SA from RA, OA and GA using serum PCT was significantly lower compared with that by synovial fluid PCT.

To the best of our knowledge, the present study is the first to simultaneously examine all three non-infectious forms of arthritis (RA, OA and GA) using serum and synovial fluid not stored under frozen conditions for >24 h. The levels of PCT in the two types of sample were compared among the four groups using the Nemenyi method. The results revealed that the levels of PCT in the serum and synovial fluid of patients with SA were significantly higher compared with those in the patients in the other three groups. No significant difference was observed in the levels of PCT among the RA, OA and GA groups. A PCT level in the two specimens of <0.5 μg/l had high specificity, PPV and NPV for the differential diagnosis of SA from RA, OA and GA; however, the synovial fluid levels of PCT <0.5 μg/l had significantly higher sensitivity than those of PCT in the serum at the same level. The results of the present study are in accordance with certain previous studies in which synovial levels of PCT were revealed to be positive indicators of SA (35) and were able to differentiate SA from OA (36). Serum PCT has previously been reported as a specific marker of SA but with low sensitivity and was considered not to be useful for the diagnosis of SA, even in association with CRP (37). Nevertheless, serum PCT has been reported to have an improved performance compared with synovial fluid PCT in the diagnosis of SA (35) and serum levels of PCT ≥0.3 μg/l have been revealed to have a specificity of 98% in the differential diagnosis of SA infection from prosthetic joint infection and a sensitivity of <35% (38–40)

Using ROC curve analysis, the present study demonstrated that the accuracy of the differential diagnosis of SA from the three types of non-infectious arthritis using synovial fluid levels of PCT was significantly higher compared with using the levels of PCT in the serum. This may be attributed to the fact that joint SA, RA, OA and GA do not cause systemic inflammation; therefore, serum levels of PCT are unable to differentiate SA from RA, OA and/or GA. Certain studies have demonstrated that low levels of PCT may result from limited inflammation or early infection (41,42). Other studies have revealed that the lack of a systemic inflammatory response causes the sensitivity of serum PCT in the differential diagnosis of infectious prosthesis to be extremely low (38–40).

In conclusion, the sensitivity and accuracy of synovial joint fluid PCT at certain levels was significantly higher than that of the serum levels of PCT. Therefore, the level of synovial fluid PCT may be used as an alternative indicator in the differential diagnosis of SA from RA, OA and GA, which may be valuable in guiding the use of antibiotics to SA. However, further studies with a larger patient sample size are required to validate this result. Further work is required to determine the optimal serum and synovial fluid levels of PCT for the differential diagnosis of SA from non-infectious arthritis.

## Figures and Tables

**Table I:** Characteristics of the SA, RA, OA and GA groups.

Characteristic	SA	RA	OA	GA
Number of cases	23	21	40	11
Male/female (n)	15/8	6/15	19/21	10/1
Average age (years)	46.6±3.6	35.0±2.2	66.2±2.6	60.8±5.6
Types and numbers of pathogenic bacteria				
*Staphylococcus aureus*	12	0	0	0
Hemolytic *Streptococcus*	5	0	0	0
*Tubercle bacillus*	2	0	0	0
*Escherichia coli*	2	0	0	0
*Streptococcus pneumoniae*	2	0	0	0

The mean ± stadard deviation is shown for the average ages. SA, septic arthritis; RA, rheumatoid arthritis; OA, osteoarthritis; GA, gouty arthritis.

**Table II:** Serum and synovial fluid levels of PCT in the knees of patients with SA, RA, OA and GA.

	SA (n=23)		RA (n=21)		OA (n=40)		GA (n=11)			
						
PCT (μg/l)	Cases	%	Cases	%	Cases	%	Cases	%	χ^2^	P-value
Serum										
<0.5	15	65.21	21	100.0	39	97.50	11	100.00	23.002	0.001
0.5–2.0	6	26.09	0	0.00	1	2.50	0	0.00		
2.0–10.0	2	8.70	0	0.00	0	0.00	0	0.00		
>10.0	0	0.00	0	0.00	0	0.00	0	0.00		
Total	23	100.00	21	100.00	40	100.00	11	100.00		
Synovial fluid										
<0.5	3	13.04	20	95.24	38	95.00	10	90.90	62.669	<0.001
0.5–2.0	9	39.14	0	0.00	1	2.50	1	9.10		
2.0–10.0	7	30.43	1	4.76	1	2.50	0	0.00		
>10.0	4	17.39	0	0.00	0	0.00	0	0.00		
Total	23	100.00	21	100.00	40	100.00	11	100.00		

PCT, procalcitonin; SA, septic arthritis; RA, rheumatoid arthritis; OA, osteoarthritis; GA, gouty arthritis.

**Table III:** Sensitivity, specificity, PPV and PNV of the serum and joint fluid levels of PCT in the differential diagnosis of septic arthritis from rheumatoid arthritis, osteoarthritis and gouty arthritis.

	Serum PCT				Synovial fluid PCT			
		
PCT (μg/l)	Sensitivity (%)	Specificity (%)	PPV(%)	NPV(%)	Sensitivity (%)	Specificity (%)	PPV(%)	NPV(%)
0.5	34.79	98.61	88.89	82.56	86.96	94.44	83.33	95.77
2.0	8.70	100.00	100.00	77.42	47.83	97.22	84.62	85.37
10.0	0.00	100.00	-	75.79	17.39	100.00	100.0	79.12

PCT, procalcitonin; PPV, predictive positive value; predictive negative value, PNV.

**Table IV:** Accuracy analysis of the serum and synovial fluid levels of PCT in the differential diagnosis of knee septic arthritis from rheumatoid arthritis, osteoarthritis and gouty arthritis.

			Asymptotic 95% confidence interval		
				
Test variables	Area	Standard error	Lower limit	Upper limit	Accuracy analysis
Serum PCT	0.761	0.064	0.635	0.887	Low to moderate
Joint fluid PCT	0.951	0.023	0.905	0.996	Higher

PCT, procalcitonin.
